# Mutually exclusive mutation profiles define functionally related genes in muscle invasive bladder cancer

**DOI:** 10.1371/journal.pone.0259992

**Published:** 2022-01-24

**Authors:** Ami G. Sangster, Robert J. Gooding, Andrew Garven, Hamid Ghaedi, David M. Berman, Scott K. Davey

**Affiliations:** 1 Division of Cancer Biology and Genetics, Department of Pathology and Molecular Medicine, Queen’s University Cancer Research Institute, Kingston, Ontario, Canada; 2 Department of Physics, Queen’s University, Kingston, Ontario, Canada; Centro Nacional de Investigaciones Oncologicas, SPAIN

## Abstract

Muscle Invasive bladder cancer is known to have an abundance of mutations, particularly in DNA damage response and chromatin modification genes. The role of these mutations in the development and progression of the disease is not well understood. However, a mutually exclusive mutation pattern between gene pairs could suggest gene mutations of significance. For example, a mutually exclusive mutation pattern could suggest an epistatic relationship where the outcome of a mutation in one gene would have the same outcome as a mutation in a different gene. The significance of a mutually exclusive relationship was determined by establishing a normal distribution of the conditional probabilities for having a mutation in one gene and not the other as well as the reverse relationship for each gene pairing. Then these distributions were used to determine the sigma–magnitude of standard deviation by which the observed value differed from the expected, a value that can also be interpreted as the ‘p-value’. This approach led to the identification of mutually exclusive mutation patterns in KDM6A and KMT2D as well as KDM6A and RB1 that suggested the observed mutation pattern did not happen by chance. Upon further investigation of these genes and their interactions, a potential similar outcome was identified that supports the concept of epistasis. Knowledge of these mutational interactions provides a better understanding of the mechanisms underlying muscle invasive bladder cancer development, and may direct therapeutic development exploiting genotoxic chemotherapy and synthetic lethality in these pathways.

## Introduction

Muscle Invasive Bladder Cancer (MIBC) is known to be heterogeneous with divergent differentiation patterns and sensitivities to therapy [1]. Whole-genome studies have identified different subgroups within MIBC, but there is some disagreement on the number of subgroups that exist [2]. However, they do agree that the subgroups contain either luminal or basal gene expression patterns [2]. Basal subtypes express proteins that are shared with bladder cancer stem cells while luminal subtypes express protein markers of terminal urothelial differentiation. In some studies, the basal and luminal subtypes show distinct responses to chemotherapy but there is more to uncover about the diagnostic and therapeutic options for MIBC patients [2].

MIBC is known to have a heavy mutation load when compared to other cancers [3, 4]. The majority of bladder cancer cases have a mutation in chromatin regulating genes, including histone methylases, demethylases and acetyl transferases, as well as members of the SWI/SNF and Polycomb repressor complexes [3]. Specific genes in these pathways that have been shown to be commonly mutated include ARIDIA, KDM6A, EP300, KMT2C, KMT2A, CREBBP, CHD7 and SCAP [3]. Chromatin modification is a mechanism that controls access of proteins to DNA. DNA repair factors and transcriptional regulators constitute two major classes of DNA binding proteins that are quite relevant to cancer formation and progression. However, the biologic and clinical significance of these mutations remains to be defined, representing a major opportunity for understanding and targeting the molecular events that drive urothelial carcinogenesis.

MIBC is also known to have an abundance of mutations in DNA damage response (DDR) genes [3]. The main DDR pathways include homologous recombination, non-homologous end joining, base excision repair, direct repair, mismatch repair, nucleotide excision repair and trans lesion synthesis. Mechanisms essential for DDR are systems that integrate DDR with the cell cycle and systems that organize and regulate DDR activity. The pathways associated with these mechanisms include chromatin remodelling, checkpoint factors, ubiquitin response, p53 and chromosome segregation [5]. On top of having very intricate interactions with these pathways, 56% of DDR proteins interact with proteins involved in a different DDR pathway [5]. DDR proteins are also known to have the ability to play different roles in different complexes within the DDR. Furthermore, the complexity of DDR continues to increase as new roles for known DDR proteins and entirely new DDR proteins are still being discovered [5].

Disruption of DDR genes is a hallmark of cancer and can be exploited therapeutically [6, 7]. One way this is done is by inducing DNA damage by means such as chemotherapy and cancer cells with hindered DDR processes are overcome by DNA damage. Another way hindered DDR is exploited is by directly targeting enzymes in DDR pathways that are essential for tumour survival by synthetic sensitivity or lethality (SSL) [8]. Synthetic lethality occurs when the combined loss of 2 functions leads to cell death, while the loss of one or the other does not. Synthetic sensitivity occurs under the same circumstances as synthetic lethality, however, instead it leads to hindered cell growth or proliferation. When synthetic sensitivity is combined with other cellular stresses it can lead to cell death. In the context of cancer, this means that one DDR process is hindered in cancer cells and another is targeted as a therapy to cause synthetic lethality or sensitivity.

Current 1st line systemic therapy for MIBC is cisplatin-based chemotherapy, which appears to substantially improve survival in a significant minority of patients [9, 10]. Mechanistically, cisplatin may exploit SSL through hindered DDR by causing inter-strand and intra-strand crosslinks in DNA [11]. ERCC2, a DDR gene, is involved in the nucleotide excision DNA repair (NER) pathway that repairs inter-strand and intra-strand cross links [11]. ERCC2 mutations are found in bladder cancer at 10–18%, but are not known to be significantly mutated in other cancers [11]. When ERCC2 mutations are present in MIBC the overall mutation rate is tripled, suggesting that this mutation does impact the tumour’s ability to repair DNA damage [11]. MIBC patients with ERCC2 mutations have been linked to near complete pathological response to cisplatin based neoadjuvant chemotherapy in some studies [12–14] though not in others. The precise mechanism that this mutation and therapy pairing suggests has yet to be confirmed. Additionally, there may be additional similar target-specific therapeutic opportunities arising from hindered DDR processes or from dysfunction in other mechanisms DDR depends on such as chromatin modification or cell cycle checkpoints.

Unfortunately, a significant proportion of MIBC patients cannot tolerate cisplatin, or acquire resistance to it [15, 16]. Once left without active alternatives, patients in this scenario now have access to 3 new and active drugs that target either immune checkpoints (i.e, PD1 and PDL1), the Fibroblast Growth Factor Receptor, or the cell surface receptor Nectin4 [17]. Despite this good news, inherent or acquired resistance to systemic therapy is a common problem for most patients in adult oncology. Thus, a broader menu of systemic therapies is needed. The high rate of mutations in CM genes and the lack of data regarding their functional significance suggests a strategy for discovering new drug targets and a new therapeutic frontier for bladder cancer.

Given the interconnectivity between DDR, CM and the cell cycle, as well as the abundance of mutations in genes in these pathways found in various MIBC cases, there are most likely relationships amongst these genes that can be described by epistasis. An epistatic relationship pattern would suggest that the outcome of a mutation in one gene would have the same outcome as a mutation in a different gene and thus display a mutually exclusive relationship between MIBC cases. The goal of this work is to evaluate statistically significant mutually exclusive relationships amongst DDR and CM genes in MIBC.

## Materials and methods

### Data sourcing and mutation pipelines

Clinical and genomic data from muscle invasive bladder cancer samples in The Cancer Genome Atlas (TCGA) was acquired through the GDC portal using the TCGA biolinks package and processed in R using R Studio. A total of 407 cases in the TCGA MIBC cohort were used in this study, where clinical data indicated the stage and histological type of the tumour. Only samples clearly listed as ‘muscle invasive urothelial carcinoma’ and histological type of stage 2 (n = 130), 3 (n = 141), or 4 (n = 136) were included.

### Mutation impact classifications and false discovery rates

Mutation impact was provided as an attribute of a mutation in the TCGA data, its classifications included high, moderate, low and modifier. These classifications represent the predicted impact a mutation will have on a proteins ability to function. Only mutations of high and moderate impact were included in our data analysis because those of low or modifier impact were not expected to have an impact on protein product. The list of DDR and CM genes was determined by combining a comprehensive list generated from three different groups [5, 18, 19]. The final step of the data refinement process was to estimate false discovery rate (FDR) for mutations in a gene for each data subgroup, using a bootstrapping technique, and implement it. The FDR for mutations in a gene is the rate at which a mutation could be found in a gene simply by chance. This rate was determined with a bootstrap simulation where the number of mutations observed in this data set were distributed among bladder genes (20,500 genes) at random 1 million times, and converged at a rate of 7%. Therefore, genes that were mutated at a rate of 7% or less could happen by chance and were not included in this analysis.

### Mutual exclusion analysis

The degree to which variants were mutually exclusive of one another was calculated using the conditional probability for each gene pairing, given single gene variant frequency. This process generated a data matrix of conditional probabilities, referred to as the CP matrix, for each data subset. Specifically, by Bayes formula we calculated: P (notA GIVEN B) = P (notA AND B) / P (B). The relationships were verified *in silico* by establishing the statistical significance of each value in the CP matrix. This process was conducted for each data set and required repeatedly randomly distributing the exact mutations amongst the cases 2000 times. The distributions of conditional probabilities were then tested for a normal distribution using shapiro-wilks test. The randomly generated CP distribution for each gene pair will be referred to as the RGCP distribution, and the mean of that distribution will be referred to as the RGCP expected value.

The RGCP distributions and values were compared to the respective CP value from the CP matrix derived from the MIBC DNA mutations in the TCGA cohort. Those that showed a statistical difference suggested the relationship did not happen at random and was in fact being selected for. Statistically significant relationships that displayed reciprocal exclusivity were visualized as a network diagram to show the interconnectivity of the mutually exclusive relationships in the mutation data. The relationships were visualized as 3 groups within the 4 data sets, *viz*. those that are outside the distribution (double line), over 2 sigma difference (single line), and those less than 2 but greater than 1.5 sigma (dotted line).

## Results

### Sample and variant classification

TCGA has mutation data for MIBC derived from 4 different mutation calling pipelines; Muse, Mutect2, Somatic Sniper, and Varscan2. Each pipeline caters to different computational and statistical methods as well as being geared towards detecting different types of mutations. To allow for the variations between these pipelines in our analysis, we chose to allocate mutation calls into three subsets: The "least conservative" subset consisted of variants identified by any pipeline; the "mid conservative" subset consisted of variants that were called by at least two of the mutation pipelines; and the "most conservative" subset only included variants that were called by all four of the pipelines. We further subdivided the data within the "least conservative" subset into variants with predicted high biological impact, and those with high or moderate predicted impact. The goal of this sub-setting was to allow the different pipelines to each contribute to our analysis, while preventing one from dominating the analysis due to a more lax variant calling routine.

**Fig 1:**
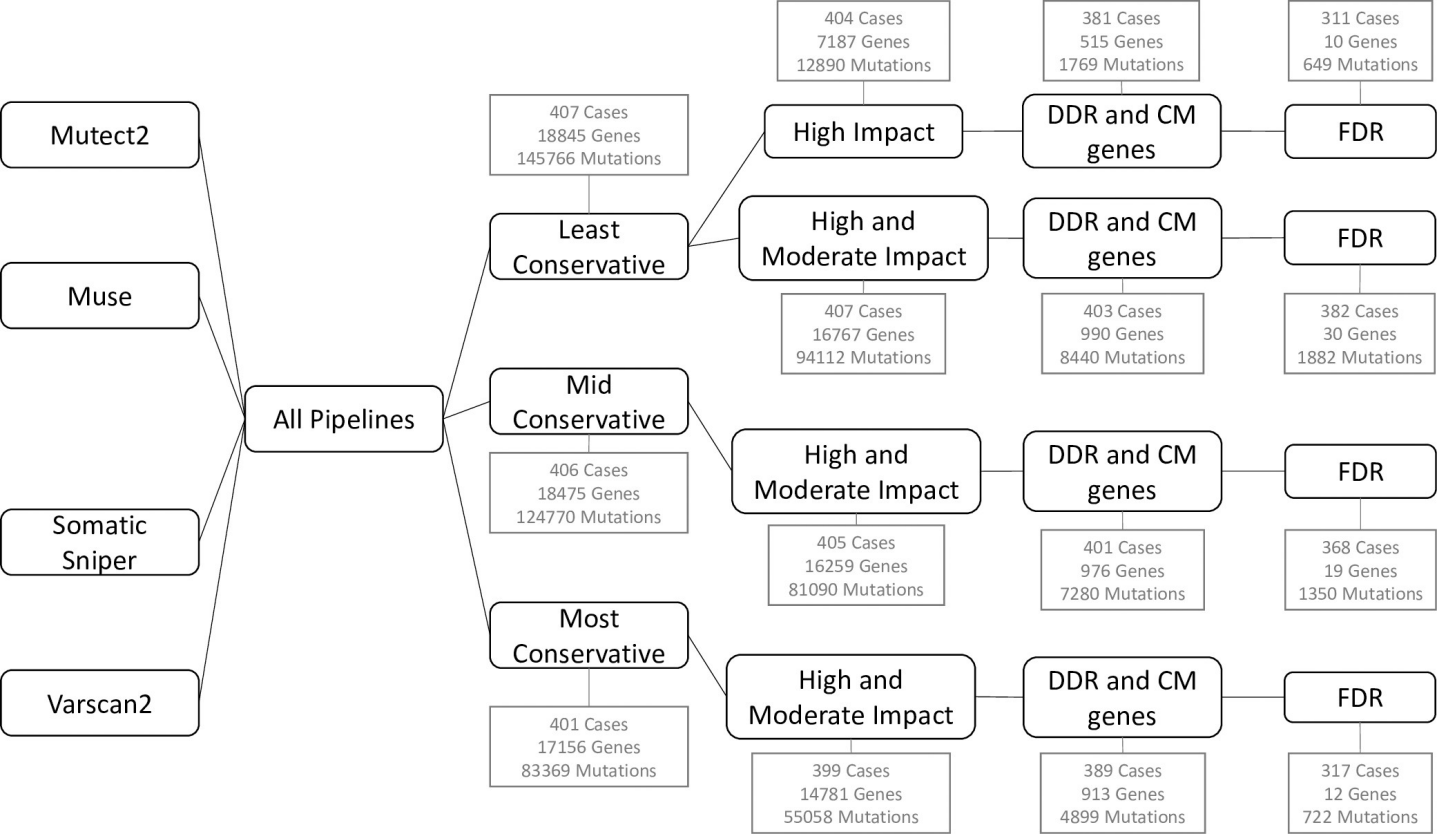
Pipeline for dataset generation. Pathways show the data selection and trimming process through each of the Lest Conservative, Mid Conservative and Most Conservative approaches. The number of cases, genes and mutations (mut) in the data at each step of the refinement process are presented in square boxes.

A summary of the number of cases, genes, and mutations for each data subset, at each step of pre-processing is presented in Fig 1. Each step in the data refinement process led to a reduction in the number of mutations and cases in the dataset, with the end result being groups with predicted high (or moderate, as indicated) impact on DDR and CM gene function, that were observed at greater than FDR rates. A list of the genes included in each subset is presented as Table 1; the complete lists of genes, along with detailed information on the total number of mutations, number of cases with a mutation, and percent of cases with a mutation is presented as S1 Table. A list of mutations called by each pipeline and sorted by sample barcode is presented as S2 Table.

**Table 1:** Summary of genes found in each of the data subsets.

Least Conservative Pipeline		Mid Conservative Pipeline	Most Conservative Pipeline
Moderate and High Impact Variants	High Impact Variants	Moderate and High Impact Variants	Moderate and High Impact Variants
ARID1A	ARID1A	ARID1A	ARID1A
ARID2			
ASH1L			
ASXL2		ASXL2	
ATM		ATM	ATM
ATR			
BPTF			
BRCA2		BRCA2	BRCA2
CDKN1A	CDKN1A	CDKN1A	
CDKN2A			
CHD6			
CHD7			
CREBBP	CREBBP	CREBBP	CREBBP
EP300	EP300	EP300	EP300
ERCC2		ERCC2	ERCC2
HUWE1			
KANSL1			
KDM6A	KDM6A	KDM6A	KDM6A
KMT2A	KMT2A	KMT2A	KMT2A
KMT2C	KMT2C	KMT2C	KMT2C
KMT2D	KMT2D	KMT2D	KMT2D
NCOR1		NCOR1	
POLQ			
RB1	RB1	RB1	RB1
SETD2			
SRCAP		SRCAP	
STAG2	STAG2	STAG2	STAG2
TP53	TP53	TP53	TP53
TRRAP		TRRAP	TRRAP
UBR5			

**Fig 2:**
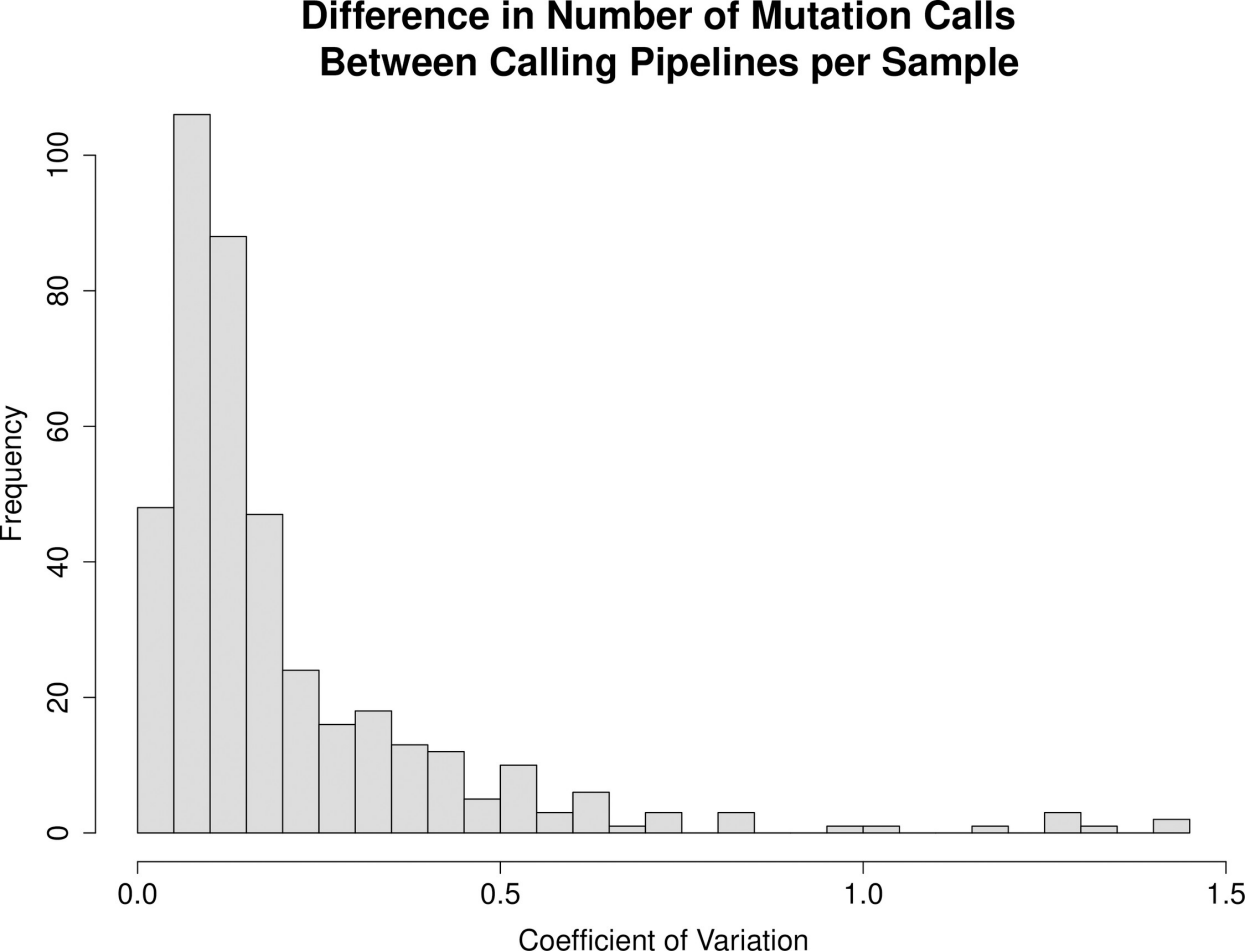
Coefficients of variation (CV) for the cases of MIBC represented in the different mutation annotation file (MAF).

**Fig 3:**
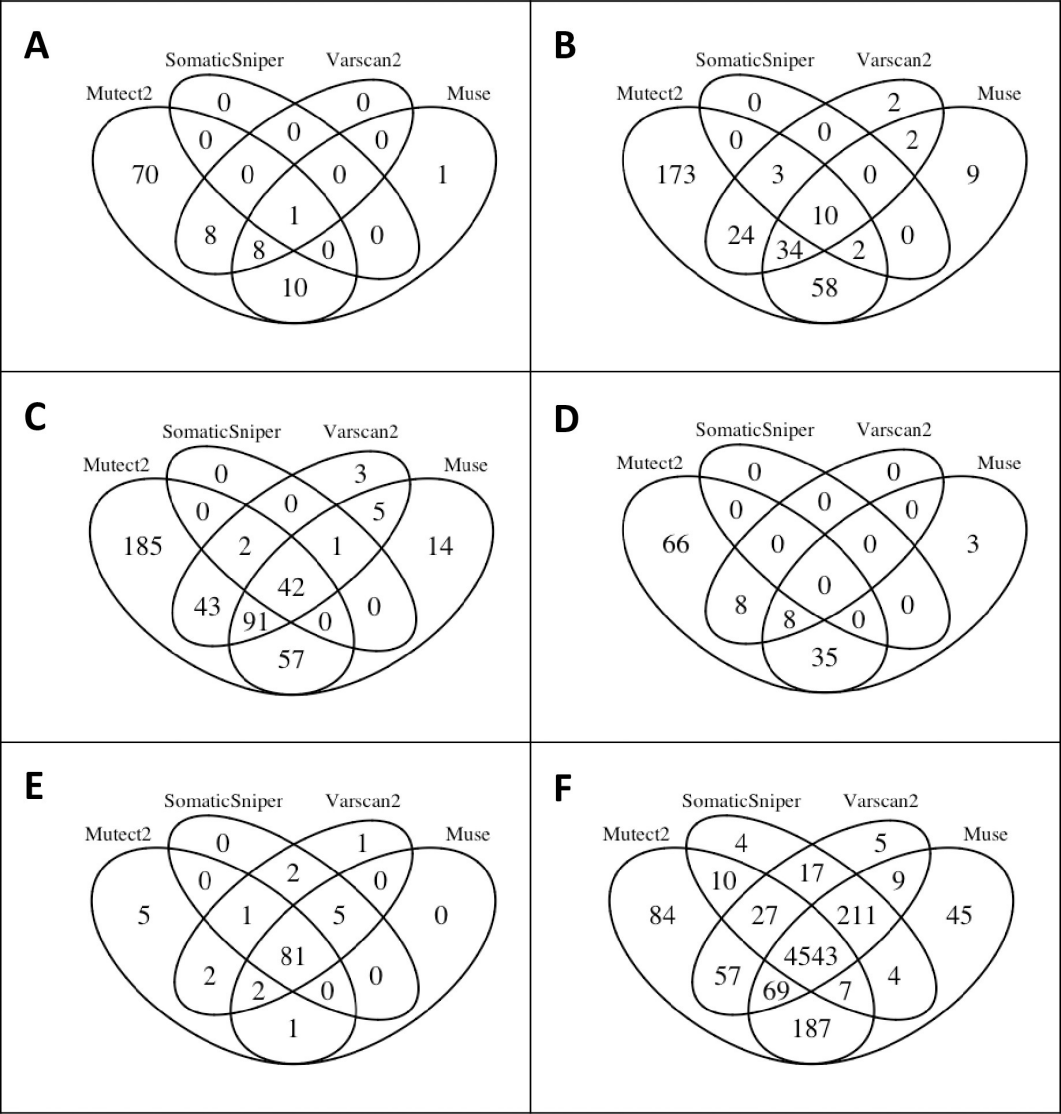
Visualization of the overlap in mutation calling between the four pipelines (Muse, Mutect2, SomaticSniper, Varscan2) mutation calling pipelines in four cases with high CV (a-d), and two cases with low CV. Sample details are: (A) TCGA-XF-A9SL-01A-11D-A391-08, CV = 1.3, 35 mean / 100 total mutation calls; (B) TCGA-K4-A83P-01A-11D-A34U-08, CV = 0.98, 125 mean /317 total mutation calls; (C) TCGA-XF-A9SI-01A-11D-A391-08, CV = 0.71, 215 mean / 450 total mutation calls; (D) TCGA-DK-A1AF-01A-11D-A13W-08, CV = 1.00, 45 mean / 120 total mutation calls; (E) TCGA-LT-A8JT-01A-11D-A364-08, CV = 0.026, 90 mean / 100 total mutation calls; (F) TCGA-DK-A6AW-01A-11D-A30E-08, CV = 0.020, 5000 mean / 5300 total mutation calls.

An initial statistical analysis showed that the average number of mutations per case varied by up to 100 mutations, with some striking outliers of over 100-fold the mutation average (Table 2). The coefficient of variation (CV = standard deviation/mean) for each case was calculated and a histogram summarizing the range of CV versus sample frequency is presented as Fig 2. To better understand variation within a specific sample, we chose four samples showing high CV and two samples showing low CV, and illustrate how each of the four pipelines called variants in these samples (Fig 3).

**Table 2:** Statistical profiles of mutation calling pipelines.

Pipeline Name	Mutect2	Muse	Somatic Sniper	Varscan2
**Total Cases**	412	411	408	412
**Total Cases Used (note 1)**	407	406	403	407
**Total Number of Mutations**	134,513	119,159	93,105	116,831
**Mean Number of Mutation**	329	292	230	286
**Mutation Number (per sample) Quartiles**	2–123	1–103	1–79	1–102
	123–229	103–200	79–147	102–196
	229–406	200–347	147–285	196–343
	406–4985	347–5077	285–4825	343–4940

Note 1: Revised for samples where the clinical data was rejected, leading to the exclusion of the sample.

### Assessment of mutual exclusivity

To determine whether the relationships between gene pairs are statistically significant and mutually exclusive, the randomly generated conditional probabilities (RGCP) distributions were plotted with the RGCP value and conditional probability (CP) value for all gene pairs. Since each of the selected RGCP distributions are normally distributed, the 68-95-99.7 (empirical) rule applies. The empirical rule means that 68% of the data in the distribution is contained within 1 standard deviation of the mean (ie. 1 sigma), 95% of the data is contained within 2 standard deviations from the mean (ie. 2 sigma, p-value of 0.05), and 99.7% of the data is contained within 3 standard deviations from the mean (ie. 3 sigma, p-value of 0.003). Since sigma is a measure of distance from the mean the percentages associated with each sigma value are easily calculated into p-values (p-value = 1-percentage). For example, the odds of randomly selecting a point in the data that has a value of 2 sigma or higher is 5% because 95% of the data has a sigma value of less than 2 sigma. Since data points with 2 sigma or higher only account for 5% of the data, they would have a p-value of 0.05 (0.05 = = 5%). As sigma increases, the percentage of data points contained at that distance decreases and so does the chance of selecting one at random, therefore the p-value also decreases.

For each gene pairing there are 2 relationships, the probability of having a mutation in one gene and not the other as well as the reverse relationship. When both relationships show statistical significance at p< = 0.05, we deem them mutually exclusive. Some gene pairings showed 2 sigma or greater for one relationship and 1.5 sigma for the other, these were included in the analysis, but they are identified by the weaker relationship. For example, if gene1 showed mutual exclusivity towards not gene2 with = >2sigma and the reverse relationship showed >1.5sigma, then the relationship will be described as >1.5sigma.

**Fig 4:**
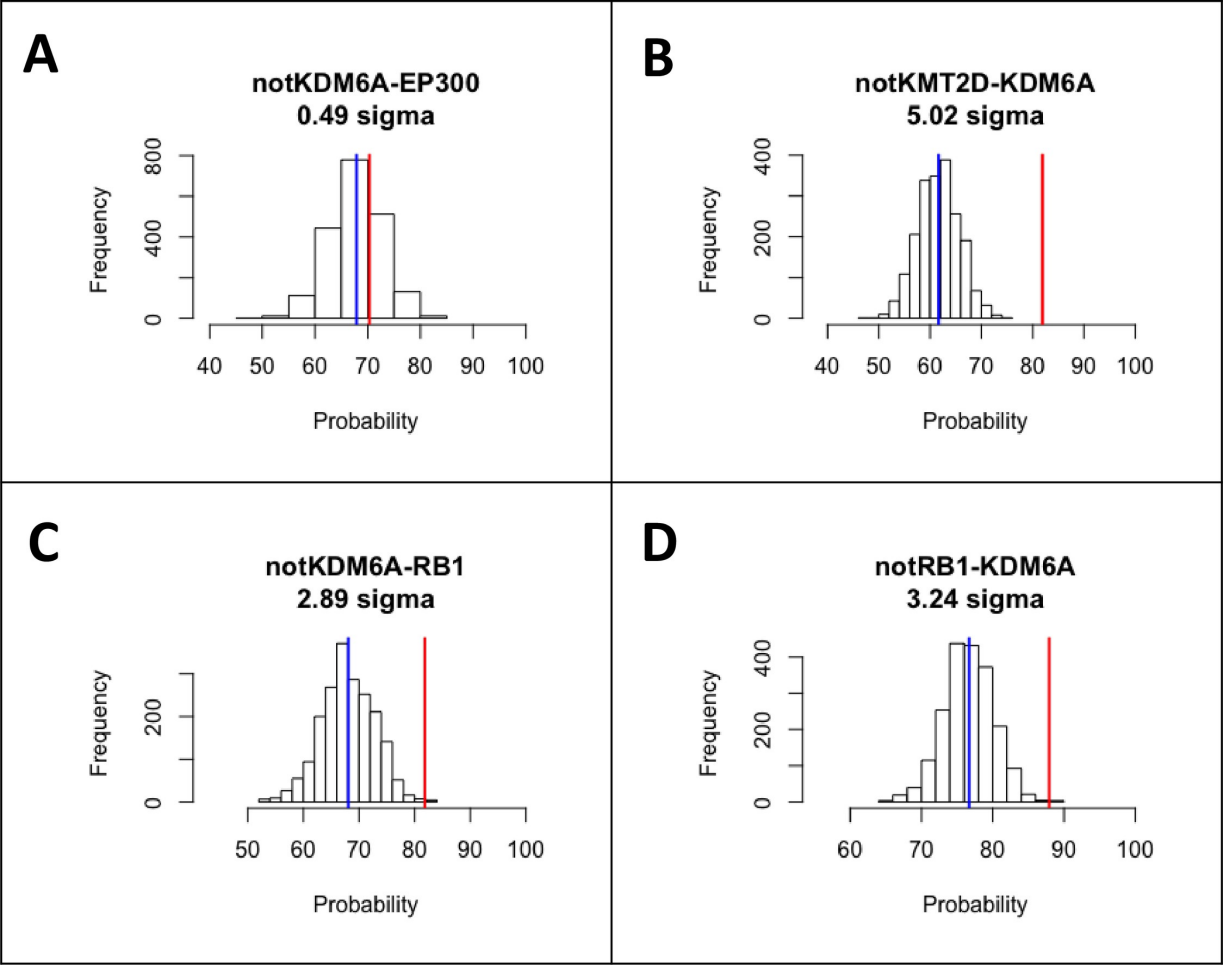
Representative differences in statistical significance of conditional probability for different gene pairs. The red bar is the mean of the RGCP distribution, and the blue bar is the value observed in the CP matrix. (A) A relationship where there is no statistically significant difference between RGCP distribution and the observed value, between the KDM6A mutation and no mutation in EP300. (B) A statistically significant relationship, where RGCP distribution for having KDM6A mutation and no mutation in KMT2D is significantly different from the observed value. (C and D) A statistically significant, bidirectional relationship, where the presence of an RB1 mutation and absence of a KDM6A mutation, and the (opposite) absence of an RB1 mutation and presence of a KDM6A mutation are both observed at frequencies significantly different from the predicted RGCP distribution.

Some pairings such as KDM6A and not EP300 did not show statistical significance, as seen by a very similar RGCP value (red) and CP value (blue) (Fig 4A). Even though this relationship demonstrates a high conditional probability (>90%) that may suggest mutual exclusivity, the result is not statistically significant because there are so few mutations in EP300, and random sampling shows the majority of cases with KDM6A are not expected to have a mutation in EP300 (also >90%). As data availability over time increases, this relationship should be further studied. Other relationships such as KDM6A and not KMT2D showed a statistically significant relationship, as demonstrated by vastly different RGCP value (red) compared to CP value (blue) (Fig 4B) despite both genes being heavily mutated. Extending the bootstrapping simulations to up to 2000 samplings allows us to predict a *p* value as small as 3x10^-7^. In the case where both genes are heavily mutated and mutated at random, the conditional probability would likely be low since they would be expected to have a higher correlation. Some gene pairs, such as KDM6A and RB1, showed statistical significance for having one mutation and not the other as well as vice versa, demonstrating a statistically significant, bidirectional, mutually exclusive relationship (Fig 4C and 4D).

### Mutation interaction network construction and analysis

**Fig 5:**
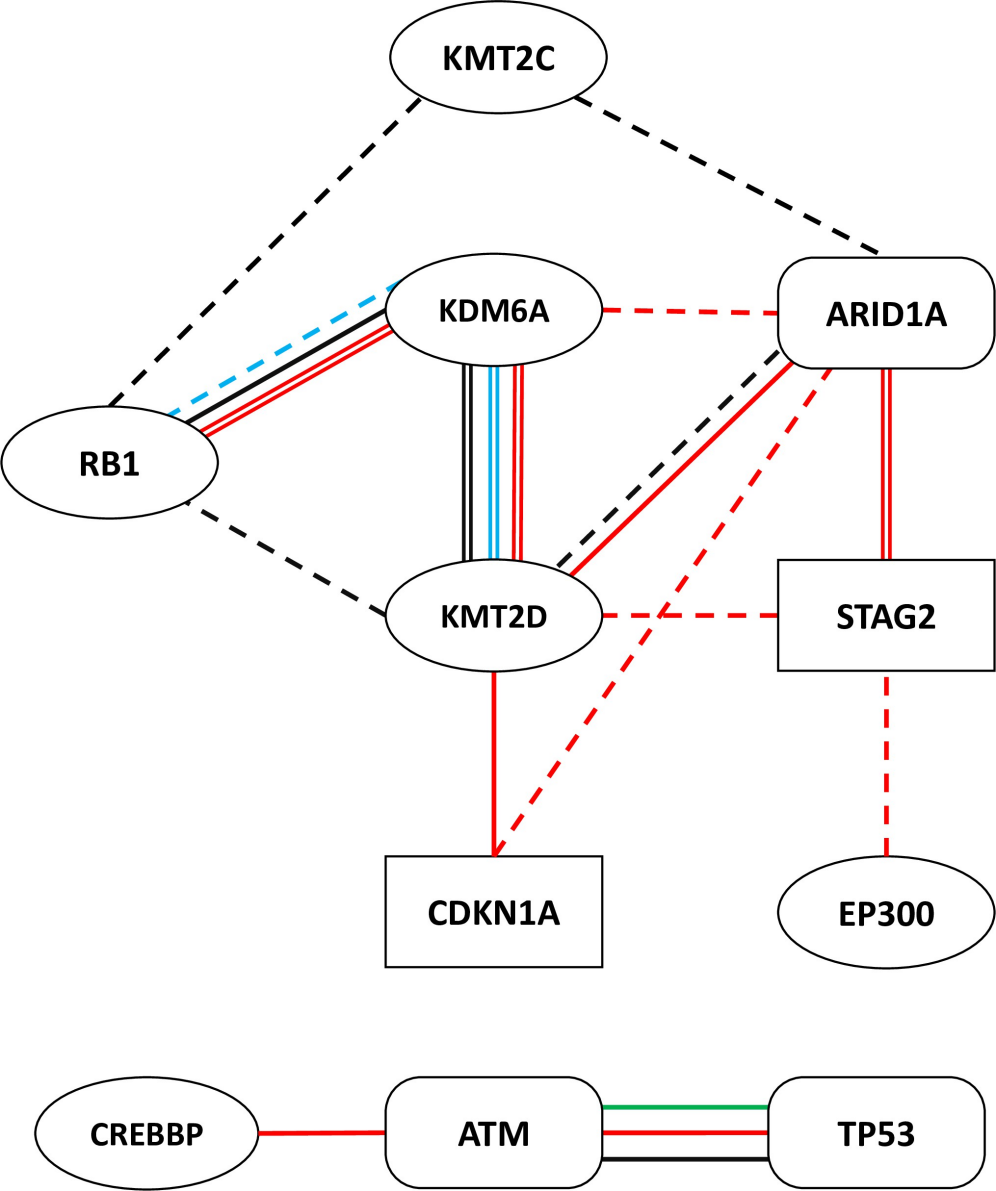
Summary of mutually exclusive relationships identified in the study. Lines between the identified genes indicate which data subset(s) the relationships were identified in, as well as the statistical strength of the observations. DDR genes are indicated by square frames, CM genes by oval frames, and genes overlapping both processes in rectangular frames with round corners. Interactions are shown for: Least High and Moderate Impact Mutations (red lines), Least High Mutations only (blue lines), Mid High and Moderate Impact Mutations (black lines), and Most High and Moderate Mutations (green lines). Statistical significance indifferences between RGCP and mean observed values is presented as: Sigma > 2.7 (double line), Sigma >2 (single solid line), Sigma >1.5 (dashed line).

We built four networks using different levels of consensus among the mutation calling pipelines, namely the most, mid, and least conservative groupings, as defined above. For each of those three groupings, we constructed a network using data that included mutations predicted to be of high or moderate impact. For the least conservative grouping only, we also generated a network using only high impact mutations; the other groupings did not have a sufficient number of high impact mutations for such analysis to be reliably completed. The network generated using the least conservative approach, supplemented with lines indicating interactions found in the other networks is presented as Fig 5. Differences and similarities in the four networks are summarized below.

Using the least conservative variant calling approach and including mutations predicted to be of both high and moderate impact includes the most cases and mutations and yielded the greatest number of mutually exclusive relationships (Fig 5 red lines). The mutually exclusive relationships between KMT2D and KDM6A, and RB1 and KDM6A showed the most statistical significance with the CP value entirely separated from the RGCP distribution and large multiples of sigma (p<0.003).

Limiting the least conservative variant calling approach to mutations predicted to have a high impact on function greatly reduced the number of variants for analysis, and yielded only a small number of statistically significant mutually exclusive relationships (Fig 5 blue lines). KMT2D and KDM6A showed a statistically significant mutually exclusive relationship where the actual conditional probability was entirely outside the RGCP distribution. Other observed mutually exclusive relationships included RB1 and KDM6A (>1.5sigma).

The use of the mid conservative variant pool, including mutation variants called by at least 2 mutation pipelines led to similar relationships found in the least conservative group (Fig 5 black lines). The most statistically significant exclusionary relationship was again found between KMT2D and KDM6A. Mutual exclusion of KDM6A and RB1 (>sigma2) was also seen in this analysis.

The use of the most conservative variant list offered the least number of significant relationships (Fig 5).

In addition to the recurrent exclusivity observed between KMT2D, KDM6A, and RB1 themselves, these genes showed additional specific exclusivities. KMT2D mutations were also exclusive with RB1, STAG2, ARID1A and CDKN1A; KDM6A mutations were exclusive with ARID1A; RB1 mutations were exclusive with KMT2C. In total, we observe 7 mutually exclusive relationships with >2sigma appearing in at least one of the networks.

KDM6A and KMT2D have previously been shown to be members of the same COMPASS complex family, alternatively called the KMT2D, MLL3/4, ASCOM complex [20]. Furthermore, mutations in either of these genes can lead to the developmental disorder kabuki syndrome [21]. To provide further experimental corroboration of their overlapping role in the context of bladder cancer, we used the TCGA bladder cancer cohort to examine the effects of mutations in KDM6A and KMT2D on a number of putative downstream targets of the complex. We identified a group of 19 genes that have been proposed to be regulated by KDM6A and KMT2D [22], and 7 genes were chosen to act as controls. In the control genes, the average expression did not change by more than 20% (up or down) when comparing either KDM6A or KMT2D to WT samples. In the experimental gene group, most (11/19) of the genes examined showed little (<20%) change in both groups; in three cases, we observed modest (<40%) change in one group, and little (<20%) change in the other. However, in five of the experimental genes examined (ABCC2, ACOX2, IL7R, ISL1, and NRG1), there was a coordinate observable decrease in expression is both KDM6A and KMT2D samples compared to WT (average decrease 48%, median decrease 46%), consistent with epigenetic downregulation. These results identify potential targets of COMPASS complexes in bladder cancer that are affected similarly by mutations in KDM6A and KMT2D and provides additional support to previous work showing that KDM6A or KMT2D loss have similar biologic consequences [20, 21].

## Discussion

In this study we uncovered statistically significant mutually exclusive relationships that are unlikely to happen by chance in the abundance of DDR and CM gene mutations in MIBC. The mutually exclusive relationship between KDM6A and KMT2D as well as the one between KDM6A and RB1 were the least likely to happen by chance and often recurred in the different subsets of the TCGA data. The mutually exclusive mutation pattern observed between these gene pairs suggests an epistatic relationship in which the mutation in one gene would have the same outcome as a mutation in the other gene. Since KDM6A connects KMT2D and RB1 mutations we hypothesize that there is a biological pathway that unites the impact of these three mutated genes and is important for the biologic fitness of bladder cancer cells.

An alternative explanation of the mutual exclusivity we have observed is that the gene pairs are synthetically lethal. In this case, exclusivity is driven by intrinsic cellular inviability, rather than by a loss of selective pressure following loss of one of the gene pair. Future functional studies in model systems will be necessary to resolve these alternatives.

An earlier study of the MIBC TCGA mutation data showed mutually exclusive relationships between CDKN2A and TP53, CDKN2A and RB1, CDKN2A and E2F3, TP53 and MDM2, FGFR3 and E2F3, and FGFR3 and RB1 [3]. Some of the relationships mentioned in the earlier study were not noted here. One reason for this discrepancy is this study focuses only on DDR and CM genes, thus excluding some genes in other functional classes, such as FGFR3. Additionally, other studies value mutually exclusive relationships on the percent of cases in which they are found, but this study valued mutually exclusive relationships based on the probability that they would not happen by chance. The current approach should better highlight gene pairings that show mutual exclusivity due to related function and excluding those that observed merely by chance.

A number of other groups have worked to develop methods for assessing mutual exclusivity of genes using publicly available genomic databases [23–25]. While two the studies presented results derived from specific cancer types, none of these works has presented results specifically addressing alterations found in bladder cancer.

### KMT2D and KDM6A

KMT2D (HGNC:7133, formerly known as MLL4 or ALR and sometimes, confusingly, MLL2) is a histone lysine methyltransferase that targets Histone 3 lysine 4 (H3K4) for monomethylation (H3K3me1), a marker of enhancer regions [20]. KMT2D is a TrxG protein and a key component of the COMPASS family complexes. COMPASS complexes that contain KMT2D are known as the KMT2D complex (or MLL3/4 Complex or ASCOM). In this complex, KMT2D provides methyltransferase activity and directly binds to the core component WRAD (consisting of WDR5, RBBP5, ASHL2 and DPY30). KMT2D plays a major role in enhancer regulation in mammalian cells, and thus contributes to many important processes such as development, differentiation, metabolism and tumour suppression [20].

KDM6A (HGNC:12637, formerly known as UTX) is a histone demethylase that targets di and tri-methylated histone H3 lysine 27 (H3K27me2 and H3K27me3, respectively) [26, 27]. It is a ubiquitously transcribed tetratricopeptide located on chromosome X, but is not affected by X-inactivation [28, 29]. This gene is linked to gene expression, embryonic development, and cellular reprogramming. KDM6A is mutated in various cancers such as breast cancer and other forms of bladder cancer [28]. In breast cancer, KDM6A was determined to be a central in the mediation of epithelial-mesenchymal transition (EMT) [28]. However, KDM6A is suggested to play many different roles in the cell as 70% of KDM6A proteins were found to co-elute with smaller complexes in breast cancer cell lines [30]. Depletion of KDM6A resulted in the silencing of HOX gene clusters by increased methylation of H3K27 in relative areas [26, 27, 31].

The KDM6A and KMT2D relationship showed the most consistent statistical significance across the different mutation data sets. These genes are connected in various ways such as being involved in the same CM complex of the COMPASS complex family and being frequently mutated in Non-Muscle Invasive Bladder Cancer (NMIBC). Additionally, mutations in either of these genes define a developmental disorder known as kabuki syndrome (KS) [21].

Not only is KDM6A associated with the KMT2D complex of the COMPASS complex family [20], but it is known to directly bind near the C terminus of KMT2D [28]. KMT2D in drosophila was shown to maintain the H3K4me1 and H3K27ac signature found at enhancers [28]. Data showing loss of KDM6A leads to reduction in H3K4me1 and H3K27ac suggests its role is to regulate the catalytic activity of KMT2D [28]. Additionally, the absence of KMT2D results in the destabilization of KDM6A as well as the collapse of the entire KMT2D complex [20].

KDM6A and KMT2D are also found to be heavily mutated in NMIBC, and mutations both genes were determined to be inactivating [29]. Interestingly, KMT2D and KDM6A mutations are positively correlated in NMIBC, they show a strong correlation in females (9% independent) and show less of a correlation in males (22% independent) [29]. In NMIBC KDM6A is more frequently mutated than it is in MIBC as well as showing a female gender bias; 74% of female patients have a mutation in KDM6A while only 42% of males had this mutation [29]. Additionally, UTY, the paralog of KDM6A is also known to be mutated in Non-Muscle Invasive Bladder Cancer (NMIBC) [29]. Of the 5 male patients with a mutation in UTY, 4 of them also had a mutation in KDM6A [29].

Recently, Lawson et al [32] have reported that the presence of alterations in KMT2D, KDM6A and a number of other cancer-related genes in microbiopsies of normal urothelium from the transplant organ donors. The authors hypothesize that this confers a selective advantage to cells harbouring such alterations over surrounding normal epithelium; it will be interesting to see whether these subpopulations of cells are precursors to cancerous lesions that develop over time.

### RB1

RB1 (HGNC:9884) encodes pRB, a commonly mutated tumour suppressor and an important regulator of the cell cycle Even though its best known role is to regulate E2F, databases suggest that pRB interacts with over 300 proteins and may play many other roles such as activating specific genes in response to apoptotic and differentiation signals [33]. Inactivation of pRB through mutation is known to interfere with cell cycle exit, promoting the cell through the cell cycle, up-regulating some of E2F target genes, and reducing senescence [33]. Interestingly, RB1 can also be compromised by mutation of proteins that impact its phosphorylation state and inactivation of pRB by phosphorylation is not functionally equivalent to the mutation of RB1 gene [33]. Along with the emerging roles of pRB, there are also emerging implications of its mutation such as up-regulation of genes needed for proliferation, genome instability, chromosome instability, aneuploidy and even metabolism [33].

The relationship between KDM6A and RB1 was present through the different subsets of the mutation data but the statistical significance of this relationship varied depending on the data subset. Under the hypothesis that mutually exclusive mutation patterns indicate the mutations have the same outcome, this relationship connects RB1 to the one between KDM6A and KMT2D. However, this relationship is not present in NMIBC since RB1 is not as frequently mutated in NMIBC as it is in MIBC [29].

One potential biological connection of RB1 to KDM6A and KMT2D is through the CM complex formed by KDM6A, KMT2D and other proteins. One of those other proteins is known as RBBP5 a key component of WRAD, and as the name suggests it binds RB1, preferably when RB1 is in an underphosphorylated state [34]. Recently, RBBP5 was found to stimulate WRAD formation, the core component of all COMPASS complexes [35]. Additionally, RBBP5 and ASH2L (another component of WRAD) interface is important for the stimulation of COMPASS catalytic activity [35]. Furthermore, KDM6A was also found to regulate many RB-binding proteins in human fibroblast cells [28]. Perhaps within the growing roles of RB1 and KDM6A, could be the regulation of the KMT2D complex.

Dividing the mutations by the number of calls each variant received causes problems when trying to assess the specificity and sensitivity of each mutation data set. While each mutation calling algorithm has its own sensitivity and specificity combining them in a way that leads to some mutations having different measures of validity leaves the entire data set with variable validity. Additionally, since the different data sets are in fact subsets of each other, finding the same relationships in the data is not a validation of the relationships. A better approach would be to test each mutation calling pipeline individually and consider the least conservative approach–to look at all the mutations collectively.

Regardless of the potential ways to improve the mathematical analysis of this data, there is a clear relationship between KMT2D and KDM6A that did not happen by chance and extends beyond the scope of altered chromatin modification in cancer and into those of known developmental disorder KS. The reasons for the non-coincidental relationship observed between KDM6A and RB1 have yet to be confirmed but the results of various studies suggest connections in more ways than one, but particularly through the critical component of all COMPASS complexes known as RBBP5. This study puts forward the importance of the COMPASS complex and all its components ranging from its active component KMT2-, to its core WRAD, and even its associated proteins KDM6A, EP300 and CREBBP, to the development and progression of MIBC. Almost all these named COMPASS complex components and associated factors are frequently mutated in MIBC and most of them are commonly mutated in many other cancers. So perhaps this mechanism of chromatin regulation and its connection to DDR and the cell cycle impacts more cancers than just MIBC.

## Supporting information

S1 Table Data subset summary. This supplementary table contains a data summary that breaks down the number of mutations and their DDR and/or CM classification. There is a summary for each data subset: Least Conservative (High and Moderate), Least Conservative (High), Mid Conservative (High and Moderate) and Most Conservative (High and Moderate).
(XLSX)

S2 Table Complete mutation list. This supplementary table contains a list of mutations that includes the HUGO symbol and the TCGA case barcode for each mutation calling pipeline (muse, mutect2, somatic sniper and varscan2) that was provided in the TCGA data.
(XLSX)
